# Dental College Commencements

**Published:** 1890-05

**Authors:** 


### Dental College Commencements.

### Ohio College of Dental Surgery.

The forty-fourth annual commencement of the Ohio College of Dental Surgery was held at the Scottish Rite Cathedral, Cincinnati, Ohio, on Wednesday, March 12th, 1890.

The number of matriculates for the session was one hundred and sixty-one.

The degree of D.D.S. was conferred on the following graduates, and an address made by James Leslie, D.D.S., Vice-President of the Board of Trustees.

| NAME. | RESIDENCE. |
|---|---|
| Frank Ehret Adams | Ohio |
| Benjamin Quict Ayres | Ohio |
| Edmund George Beal | Pennsylvania |
| Michael August Becker | Ohio |
| Harry Ruthven Bell | Ohio |
| Willie Morton Bogue | Indiana |
| Carl Edward Booren | Minnesota |
| Homer C. Brown | Ohio |
| Stephen Abner Brown | Pennsylvania |
| Dalton D. Cunningham | Pennsylvania |
| Charlie C. Carle | Ohio |
| William James Crampton | Canada |
| Fred. Leslie Cauch | California |
| James Hamilton Clark | Ohio |
| Herbert Everett Crocker | Connecticut |
| Isaac Stanton Carter | West Virginia |
| Jacob Jones Donaldson | Pennsylvania |
| Sidney Allen Donaldson | Kentucky |
| Max M. Elbe | Kentucky |
| Adolph Eicke | Germany |
| Bartlett Joseph Emery | Ohio |
| Hampton Geiger | Ohio |
| Hugh Peebles Gillispy | New York |
| W. Ohmer Girardey | Ohio |
| William Clifford Griffith | Ohio |
| Daniel Ephram Hartwell | Indiana |
| W. Howard Hayden | Ohio |
| Charles Lee Hill | Ohio |
| William Henry Houser | Ohio |
| Abraham Gantz Herr | Michigan |
| Virgil Newton Jones | West Virginia |
| William Irwin Jones | Ohio |
| Isaac Edward Josephis | Pennsylvania |
| Atsuhiko Katayama | Japan |
| Samuel Dora Laughlin | Kentucky |
| James William Leahy | Ohio |
| Allen Joseph Lee | Kentucky |
| Richard Morgan, Jr. | Missouri |
| Fermine Engle Morgan | Ohio |
| John Gilpin Macy | Ohio |
| Russell McClanahan | Indiana |
| Horace Edwood McClelland | Ohio |
| Thomas Harris McClure | Pennsylvania |
| James Elmore Nichols | Canada |
| James Gilmore Parr | Ohio |
| Clarence Courtland Pollitt | Kentucky |
| J. Hardin Pollock | Ohio |
| Adam Burr Purdy | Canada |
| Elmer W. Ream | Indiana |
| Charles Lawrence Rose | Minnesota |
| Frank Stanley Rose | Canada |
| Moritz Carl Saul | Germany |
| Franklin N. Seeley | Ohio |
| Lewis S. Seeley | Ohio |
| James Wilber Shane | Ohio |
| Charles Frederick Shober | Canada |
| J. August Shober | Canada |
| Albert Sidener | Ohio |
| James A. Sinnett | Ohio |
| Moses Shobe Smith | Kansas |
| Claude Henry Thompson | Ohio |
| William H. Wernett | Ohio |
| Lorne Wilkie | Michigan |
| Edwin John Witherspoon | Michigan |
| Bayard Alvin Wright | Pennsylvania |
| J. F. Cope | Pennsylvania |

TOTAL 66.

Prizes were given as follows: Best general examination, B. A. Wright, of Pennsylvania; best attainment in operative dentistry, Russell McClanahan, of Indiana; best attainment in prosthetic dentistry, A. G. Herr, of Michigan.

### University of Maryland--Department of Dental Surgery.

The eighth annual commencement of the Department of Dental Surgery of the University of Maryland was held at the Lyceum Theater, Baltimore, Maryland, on Wednesday, March 19th, 1890.

The number of matriculates for the session was one hundred and thirty-five.

The degree of D.D.S. was conferred on the following graduates by Hon. S. Teackle Wallis, L.L.D., Provost of the University:

| NAME. | RESIDENCE. |
|---|---|
| David Aiken | South Carolina |
| J. Maurice Ayer | North Carolina |
| John R. Berry | Virginia |
| William E. Beachley | Maryland |
| William C. Boswell, Jr | Maryland |
| Charles T. Breedlove | Arkansas |
| Wm. S. Brown, Jr | South Carolina |
| Oscar Frank Byrd | Virginia |
| Stuart Cassard | Maryland |
| William H. Collins | Virginia |
| St. George T. Craig | Kentucky |
| Wray Wythe Davis | Virginia |
| L. Thornwell Emerson | Brazil, S. A |
| Charles Felker | New York |
| Henry J. Fenn | New York |
| Isaac Watts Furman | New York |
| Harvey E. Glatfelter | Pennsylvania |
| Charles Carroll Graham | Texas |
| J. William Graves | Missouri |
| N. Robert Hubbard | Pennsylvania |
| Aaron Victor Huntzbery | Maryland |
| W. Spry Hurlock | Maryland |
| Robert L. Harley | South Carolina |
| William A. King | Canada |
| Daniel O. M. LeCron | Iowa |
| John L. Luke | Virginia |
| J. Henry Marchant | Virginia |
| Otis H. McDonald | Georgia |
| James Elijah McNeal | Maryland |
| Thomas P. Mayer | Pennsylvania |
| Charles Mezger, Ph. G | Maryland |
| Laurent S. Mitchell | New Jersey |
| William W. Morgan | New Jersey |
| Charles Grant Meyers | New York |
| George Harkness Perrin | Canada |
| William A. Pressley | North Carolina |
| Charles B. Renoe | Missouri |
| Halsey G. Steer | New York |
| Sylvanus Claude Sykes | Maryland |
| F. Foster Todd | Maryland |
| Charles B. Whelpley | Canada |
| Charles Edmund Wogan | Pennsylvania |
| George Woolsey | California |
| Clarence A. Wright | New Hampshire |

### Columbia University—Dental Department.

The third annual commencement of the Dental Department of the Columbia University was held at Albaugh's Opera House, Washington, District of Columbia, on Thursday, March 20th, 1890.

The number of matriculates for the session was eleven.

The degree of D.D.S. was conferred on the following graduates:

| NAME. | RESIDENCE. |
|---|---|
| Stephen B. Cossin | Dist. of Columbia |
| Jessie Koppeler | England |
| C. M. O. Seary | District of Columbia |
| John S. Reed | District of Columbia |
| T. B. Stubblefield | District of Columbia |

### Baltimore College of Dental Surgery.

The fiftieth annual commencement of the Baltimore College of Dental Surgery, was held at Ford's Opera House, Baltimore, Md., on Thursday, March 20th, 1890, at 2 o'clock P. M.

The number of matriculates for the session was one hundred and sixty-one.

The degree of D.D.S. was conferred on the following graduates by Prof. R. B. Winder, Dean of the Faculty:

CLASS OF '90, B. C. D. S.

| NAME. | RESIDENCE. | NAME. | RESIDENCE. |
|---|---|---|---|
| J. B. Archer | California | Noyes J. Beemer | New York |
| J. P. Bayon | Louisiana | J. H. Benton, M.D. | North Carolina |
| T. A. Beal | Pennsylvania | H. J. Burkhart | New York |
| A. L. Beard | Wisconsin | J. Clarence Busey | Maryland |
| A. Foley Butler | Maryland | J. E. Dugger | North Carolina |
| J. F. Davidson | Georgia | J. E. Freeland | North Carolina |
| C. R. Diffenderffer | Maryland | B. D. Friedenwald | Maryland |
| H. A. Donaldson | District of Columbia | A. H. Goodwin | New Brunswick |
| F. M. Hampton | Alabama | W. E. Holt | New York |
| W. H. Hargrave | North Carolina | M. H. Hunter | North Carolina |
| J. H. Harrington | Kentucky | W. G. Jankens | Virginia |
| C. A. Hews | Maine | L. D. Kelley | Maryland |
| R. L. Lamar | Georgia | H. C. McBrair | New York |
| J. H. London | North Carolina | F. L. McGraw | New York |
| T. M. Linn | Iowa | W. L. Minson | Georgia |
| W. H. Mathews | New Jersey | C. W. Minton | Pennsylvania |
| W. W. Niles | New York | J. E. Parker | California |
| J. A. Noll | New York | J. T. Parker | California |
| H. Ordonez | South America | L. R. Pennington | Delaware |
| B. M. Oxley | Nova Scotia | G. A. Potter | New York |
| W. S. Pyle | Maryland | H. N. Richardson | West Virginia |
| F. M. Readio | Massachusetts | J. E. Rutledge | South Carolina |
| R. W. Reese | North Carolina | W. J. Shelby | Maryland |
| E. H. Reed | Georgia | E. O. Sims | Florida |
| F. E. Smith | Connecticut | W. M. Spaulding | Minnesota |
| D. T. Smithwick | North Carolina | W. Sproul | Canada |
| A. B. Soule | Vermont | H. M. Staire | California |
| W. H. Spangler | Maryland | R. W. Steuart | Indiana |
| F. C. Street | Maryland | E. H. Webb | South Carolina |
| A. G. Strickler | Pennsylvania | E. T. White | New Zealand |
| J. C. Sutherland | Maryland | J. F. Wilson | South Carolina |
| A. Viets | Nova Scotia | U. V. Withee | Maine |

### Pennsylvania College of Dental Surgery.

The thirty-fourth annual commencement exercises of the Pennsylvania College of Dental Surgery were held at the Academy of Music, Philadelphia, Penn., on Friday evening, February 28th, 1890, at 8 o'clock.

The address to the graduates was delivered by Prof. J. Ewing Mears, A.M., M.D.

The number of matriculates for the session was one hundred and eighty-six.

The degree of D.D.S. was conferred on the following graduates by I. Minis Hayes, M.D., President:

| NAME. | RESIDENCE. |
|---|---|
| R. W. Allan | New York |
| G. C. Anthony | Pennsylvania |
| A. S. Atkinson | England |
| Rupert G. Beale | Pennsylvania |
| Edw. V. Beardsley | New York |
| J. S. Birdsong | Mississippi |
| R. W. Berthel | Pennsylvania |
| R. T. Brown | Pennsylvania |
| J. H. Burton | Delaware |
| A. H. Butterfield | New York |
| R. C. Carr | Canada |
| Perry R. Chance | Ohio |
| Lydia C. Clare | New York |
| H. H. Colehower | Pennsylvania |
| E. S. Cornell | New York |
| Chas. O. Dager | Pennsylvania |
| S. S. Davis | Pennsylvania |
| I. H. Deacon, D.D.S | Pennsylvania |
| Guillermo Esguerra | U. S. Columbia |
| Geo. A Ewell | Massachusetts |
| F. W. Farmer | New York |
| Archie G. Fee | Minnesota |
| Felix Fischler | Germany |
| Benj. Frontz | Pennsylvania |
| T. P. Gardner | Kentucky |
| M. H. Glocker | Pennsylvania |
| C. F. Gorham | Cauada |
| B. C. Hannington | Canada |
| M. P. Harrington | Canada |
| W. D. Hays | Pennsylvania |
| O. L. Hertig | Pennsylvania |
| H. S. Hollenback | Pennsylvania |
| Mortimer Hopkins | New York |
| W. J. Hottenstein, M.D | Pennsylvania |
| E. K. Hottenstein, M.D | Pennsylvania |
| H. F. Jordan, M.D | Mississippi |
| Gust. Joachim | Germany |
| J. D. Kendig | Pennsylvania |
| Geo. W. Kesel | Pennsylvania |
| B. M. Knight | Virginia |
| J. Orr Kyle | Ireland |
| W. B. Lake | Pennsylania |
| A. A. Lay | New York |
| Edmund Lord | Pennsylvania |
| C. H. Lorance | New Jersey |
| Carl Lorenz | Germany |
| H. T. Martin | Pennsylvania |
| J. W. Meisgeier | Pennsylvania |
| M. F. Miller | New York |
| Martha B. Moore | Pennsylvania |
| J. W. A Musgrove | Canada |
| Geo. S. Nason | Nebraska |
| Margaret Neilson | New Jersey |
| Porter S. Oakley | New York |
| W. E. C. Orr | Pennsylvania |
| D. W. Peterson | Canada |
| G. H. Phelps | Massachusetts |
| T. A. Powell | Pennsylvania |
| G. H. Rabenold | Pennsylvania |
| R. L. Ramsey | North Carolina |
| J. D. Ribeiro, M.D | Brazil |
| E. V. Rice | Minnesota |
| Benj. F. Rose | Pennsylvania |
| W. D. Scholl | Pennsylvania |
| E. V. Sheerar | New York |
| G. H. Simmerman | New Jersey |
| E. H. Slocum | New Jersey |
| I. V. Smith | Virginia |
| A. J. Timmons | Maryland |
| Santiago Uribe | U. S. Columbia |
| J. S. Voegtlen | New Jersey |
| F. D. Wattson | Pennsylvania |
| A. G. Weber | Cuba |
| Ignatius R. Weber | Cuba |
| J. F. Wessels, Jr | Pennsylvania |
| H. C. Wetmore | Canada |

### University of California.

The eighth commencement exercises of the College of Dentistry of the University of California were held at Odd Fellows' Hall, San Francisco, Wednesday, November 13th, a 8 o'clock P. M.

Number of matriculates was fifty-one.

The degree of D.D S. was conferred on the following graduates by Horace Davis, A.B , President of the University :

| NAME. | RESIDENCE. |
|---|---|
| Milton Ross Gambitz | California |
| Benjamin M. Gunzburger | California |
| William Alexander Meyer | California |
| Warren Guice Mobley | California |
| Dorr Nash | California |
| Andrew John Powell | California |
| Edward William Pratt | California |
| Frederick Courtland Sutliff | California |
| Arthur Henry Wallace | California |

### Missouri Dental College.

The twenty-fourth annual commencement exercises of the Missouri Dental College, were held in connection with those of the St. Louis Medical College, at Memorial Hall, St. Louis, Mo., on Thursday evening, March 13th, 1890.

The number of matriculates for the session was seventy-eight.

The degree of D.D.S. was conferred on the following graduates by Prof. W. H. Eames, D.D.S., of the Faculty:

| NAME. | RESIDENCE. | NAME. | RESIDENCE. |
|---|---|---|---|
| William H. Auer | Missouri | Paul W. Keller | Missouri |
| Thomas T. Baker | Illinois | Frank M. Lowry | Illinois |
| Walter M. Bartlett | Missouri | Marcus A. Mace | Illinois |
| Edward W. Bear | Missouri | Peter H. Morrison | Missouri |
| Albert G. Bowman | Louisiana | Lorenzo A. Naumann | Missouri |
| Frank H. Caughell | Missouri | Charles W. Ott | Kansas |
| Wm. A. M. Cumming | Illinois | Theo. L. Pepperling | Missouri |
| John E. Deggendorf | Missouri | Thomas N. Perrine | Illinois |
| Warden B. Dennis, Jr | Illinois | Harry W. Pierce | Indiana |
| Peter H. Eisloeffel | Missouri | James H. Prathero | Nebraska |
| Henry D. Field | Missouri | Edward Scrantz | Missouri |
| John W. Forden | Illinois | Benjamin O. Stevens | Missouri |
| Jos. Carter Goodrich | Missouri | David R. Taggart | Illinois |
| John J. Greer | Missouri | Thomas E. Turner | Missouri |
| Edwin C. Hammen | Missouri | Edgar M. Whitsett | Missouri |
| Guilford B. Houston | Missouri | Frederick V. Waldron | Pennsylvania |
| Frank A. Kimler | Illinois | Francis W. Willard | Illinois |

### Indiana Dental College.

The eleventh annual commencement exercises of the Indiana Dental College were held at Plymouth Church, Indianapolis, Ind., on Thursday evening, March 6th, 1890.

The address to the graduates was delivered by Dr. John R. Clayton.

The number of matriculates for the session was eighty.

The degree of D.D.S. was conferred on the following graduates by Seneca B. Brown, M.D., D.D.S., President of the Executive Board:

| NAME. | RESIDENCE. | NAME. | RESIDENCE. |
|---|---|---|---|
| Ellard D. Bailey | Indiana | N. F. Hazlett | Indiana |
| B. C. Brimmacomb | Ontario | George E. Hunt | Indiana |
| Albert H. Brown | Indiana | Burton W. Jones | Michigan |
| Thomas E. Coffin | Indiana | Eldridge H. Keith | Wisconsin |
| A. Morris Coffin | Indiana | W. Wallace Mungen | Indiana |
| Herbert L. Cormican | Wisconsin | Charles A. Rowand | Indiana |
| Leo A. Cox | Minnesota | Roscoe W. Reese | Indiana |
| Thomas H. Davidson | Indiana | Burt. W. Sober | Illinois |
| Charles E. Ervin | Indiana | George W. Tainter | Missouri |
| Edward G. Fry | Indiana | Edward R. Tripp | Wisconsin |
| Robert B. Gentle | Indiana | Will L. Tevis | Indiana |
| Frank B. Gonzales | Indiana | George W. Thompson | Indiana |
| Everett H. Green | Minnesota | Harry B. Tucker | Indiana |
| John H. Hess | Indiana | | |

### New York College of Dentistry.

The twenty-fourth annual commencement of the New York College of Dentistry was held at Chickering Hall, New York City, on Tuesday evening, March 11th, 1890.

The number of matriculates for the session was two hundred and forty-seven.

The degree of D.D.S. was conferred on the following graduates by William T. LaRoche, D.D.S., Vice-President of the Board or Trustees:

De Lancy Bradner Armstrong.

Francisco Genaro Bruno.

Alfred Bartels.

Edward Howard Babcock.

George William Baab.

Edwin Betts.

Manly Collard Burns.

Charles Edwin Baldwin.

Joseph Robert Bomann.

Lewis Clarence Baldwin.

James Dawson Cook.

Homer Cecil Croscup.

Frank Tittsworth Clawson.

William Greer Clark.

Alfredo Duran.

Alfred Williston Davisson.

William Henry Draper.

Wilber Stanton Dailey.

Arthur Eugene Davenport.

Myron James Dixon.

William Romanoff Dunster.

Lewis Everett Estler.

Edward Eberle.

James Samuel Eckley.

George Henry Euler.

Frank Wroe Eichhorn.

George Alfred Fournier.

Charles Andrew Fones.

Frederick Ernest Adolph Faber.

Ossian Lucerne Field.

Andrew Graham.

Jose Guillermo Guetierrez.

Ferdinand Walter Griebel.

Francis Gray.

Ellis Allen Grossmann.

George Alfred Hull.

Chester Thomas Hustis.

Thaddeus Pomeroy Hyatt.

John Julien.

Harry Colby Kahlo.

Charles Brodhead Kenney.

Roswell DeLancey King.

Louis Wixon Kennard.

Hurbert Aria Lewis.

Chas. Fredrik Josef Lundgren.

James Buckley Locherty.

Charles Carroll Linton.

Oscar Benjamin Lopez.

William John Macom.

Otto Mattes.

Fred Harry Martin.

Ferdinand Miller.

William Hopkins Merritt.

Edward O'Neill.

Nelson Mangam Pattison.

Stephen Palmer.

Ellis Frank Potter.

George Bender Poole.

Louis Simon Rosenstiel, Jr.

Frank Aloysius Ryan.

William Arja Rowlands.

Harry Johnson Sinclair.

Julius Joseph Stier.

Paul Schoenemann.

William Polly Sullivan.

Walter Clinton Spooner.

Henry Augustin Spang.

William Augustus Strong.

Frank Russell Stillman.

Arthur Hamilton Smith.

William John Schreiber.

Thomas Andrew Sproat.

Henry Paul Travers.

Frank Seymour Van Nostrand.

John William Van Doorn.

William Leslie Weed.

Frank Edmund Weber.

John Raymond Westervelt.

Horace Neverton Warren.

Julius Zietz.

### Philadelphia Dental College.

The twenty-seventh annual commencement exercises of the Philadelphia Dental College were held at the Academy of Music, Philadelphia, Pa., on Thursday, February 27th, 1890, at 8 P. M.

The number of matriculates for the session was two hundred and seventy-three.

The degree of D.D.S. was conferred on the following graduates by the president of the board of trustees:

| NAME. | RESIDENCE. |
|---|---|
| John L. Allen | Maine |
| William M. Baker | Ohio |
| Henry A. Bary | Pennsylvania |
| John E. Banner | North Carolina |
| Charles W. Banner | North Carolina |
| Lot Bell | Pennsylvania |
| Robert A. Baldwin | Massachusetts |
| Frank N. Bidwell | Connecticut |
| Merle D. Bishop | Pennsylvania |
| John E. W. Bissell | New York |
| Edgar T. Boyd | Egypt |
| George E. Boynton | Maine |
| Everet C. Borland | Pennsylvania |
| Charles A. Brennan | Pennsylvania |
| H. C. Barnhart | Iowa |
| George B. Cameron | Canada |
| John M. Campbell | Wisconsin |
| William A. Capon | Canada |
| Irvin A. Chew | Ohio |
| Edwin N. Clark | Rhode Island |
| Egbert A. Clark | Canada |
| Kendall L. Cleaves | Maine |
| Amasa F. Conwell | Massachusetts |
| Claude A. Conover | New Jersey |
| Fred. W. Conover | Iowa |
| Chas. A. Coppinger | New Jersey |
| Charles A. Darling | Montana |
| Oscar Davis | Australia |
| Charles Denison | New York |
| Bartholomew Dolan | Pennsylvania |
| A. James Edwards | Canada |
| Ephraim B. Fast | Pennsylvania |
| Constantine T. Felt | Pennsylvania |
| M. W. Flynn | Massachusetts |
| Thomas J. Frazier | California |
| Harry V. Frink | Pennsylvania |
| Geo. N. Goldthwait | Massachusetts |
| Alice A. Graham | Pennsylvania |
| Edwin R. Green | Pennsylvania |
| Max Greenbaum | Pennsylvania |
| George A. B. Hall | British Columbia |
| Mark M. Ham | Georgia |
| Wm. Booth Hentz | Brazil |
| W. A. Heckard | Ohio |
| Frank L. Hindle | New Jersey |
| Oliver P. Henderson | California |
| Charles W. Hodgman | Illinois |
| Wm. C. Hoffman | Pennsylvania |
| Edwin E. Horning | Canada |
| Percy E. Howe | Maine |
| Lynn Hoopes | Pennsylyania |
| George C. Hubbel | Connecticut |
| S. Grant Hunsberger | Pennsylvania |
| J. Fred Huntington | New York |
| Edwin B. Hurd | Ohio |
| William H. Jones | New York |
| Alvah Johnston | Pennsylvania |
| Elis'h A. Kirkpatrick | Montana |
| Robert E. Kyle | Wisconsin |
| M. F. M. Kelly | Georgia |
| Albert E. Lester | Australia |
| George E. Locke | New York |
| Maria Lasser | Pennsylvania |
| Daniel Marks | New York |
| Henry A. Mansfield | Maine |
| Herbert M. Metcalf | Missouri |
| William B. Minor | New York |
| Frank P. Miller | New York |
| W. Haliburton Morse | Canada |
| Henry A. Moore | New York |
| Charles H. Morris | Connecticut |
| Robert O. Moorhead | Pennsylvania |
| Albert L. Morrison | Rhode Island |
| Jos. B. McCaskey, Jr. | Pennsylvania |
| J. M. McLachlin | Canada |
| Jesse B. McBride | Missouri |
| J. Sinclair McKay | Canada |
| Wm. H. McKerrall | Ohio |
| Henry McManus | Connecticut |
| David A. MacMullan | California |
| Frank Noville | Ohio |
| Frank H. B. Parke | Ohio |
| Charles G. Pease | New York |
| Walter R. Peters | Delaware |
| Frank L. Platt | California |
| Lawford G. Pullen | Canada |
| Percival A. Purdy | Washington |
| Charles A. Reed | New Jersey |
| Edward E. Richer | Pennsylvania |
| Charles B. Rider | Pennsylvania |
| C. C. Richardson | Canada |
| Felix V. Riviere | Louisiana |
| William Lee Ryder | Georgia |
| Archibald E. Rykert | Canada |
| Harry E. Ruffner | Pennsylvania |
| Albert E. Sangster | Canada |
| Elmer Sigler | New Jersey |
| Andrew J. Senecal | New York |
| Albert A. Shaw | Canada |
| Sheridan Slocum | New York |
| Frederick M. Smith | New Jersey |
| George F. Smith | Canada |
| D. Sherman Smith | Pennsylvania |
| H. F. A. Schmitt | Pennsylvania |
| Walter Swazey | Massachusetts |
| Richard Stites | Kentucky |
| John W. Thomas | Pennsylvania |
| Edwin G. Thompson | Canada |
| Wm. H. Thompson | New Hampshire |
| Herbert E. Vaughn | New York |
| Paul Voigt | Pennsylvania |
| Herbert L. Wheeler | Massachusetts |
| Lewis A. Wilbur | Rhode Island |
| Samuel C. Wilson | Canada |
| J. H. Young | Canada |
| J. L. Young | Canada |

### Vanderbilt University—Department of Dentistry.

The eleventh annual commencement exercises of the Department of Dentistry of Vanderbilt University were held at Watkins Institute, Nashville, Tenn., on the evening of February 25th, 1890.

The number of matriculates for the session was one hundred and one.

The degree of D.D.S. was conferred on the following members of the graduating class:

| NAME. | RESIDENCE. |
|---|---|
| R. P. Anderson | North Carolina |
| J. E. Andrews | Arkansas |
| W. D. Anderson | Mississippi |
| A. P. Brown | Alabama |
| D. F. Bentley | New York |
| Crockett Campbell | Louisiana |
| J. W. Combs | Texas |
| Jas. H. Downie | Michigan |
| J. E. Dugger | Louisiana |
| Arthur H Douglas | Michigan |
| C. N. Folse | Louisiana |
| J. L. Fox | New York |
| A. E. Frazier | North Carolina |
| F. M. Gillespie | Alabama |
| Wm. H. Guess | Mississippi |
| C. L. Gillespie | Tennessee |
| Earnest L. Hestle, | Alabama |
| E. J. Harrison | Louisiana |
| W. C. Houston | North Carolina |
| J. L. Howard. | California |
| C. C. Hermann | Indiana |
| L. V. Hightower | Alabama |
| Jas. W. Jones | Alabama |
| Frank C. Johnson | Tennessee |
| N. C. Leonard | Tennessee |
| F. U. Meadows | Louisiana |
| Robert E. Morrow | North Carolina |
| Wm. F. Price | Virginia |
| J. W. Peden | Tennessee |
| J. C. Pearson | Alabama |
| J. W. Robson | Texas |
| D. F. Robinson | Alabama |
| T. W. Smith | Texas |
| F. W. Smith | Michigan |
| C. S. Shields | Alabama |
| T. M. Stowers | Mississippi |
| H. H. T. Segrest | Mississippi |
| W. H. Stokes | Mississippi |
| Chas. H. Taylor | Tennessee |
| L. B. Torrence. | Missouri |
| E. J. Tucker | North Carolina |

### Central Tennessee College—School of Dentistry.

The fourth annual commencement exercises of the School of Dentistry of Meharry Medical Department of Central Tennessee College, were held in connection with those of the medical and pharmaceutical departments, at Masonic Theatre, Nashville, Tenn., on Thursday, February 27th, 1890.

The number of matriculates for the session was seven.

The degree of D.D.S. was conferred on the following graduates of the dental class by Dr. J. Baden, President of the Faculty:

| NAME. | RESIDENCE. |
|---|---|
| D. Green Ferrell | Texas |
| S. James Watkins | Tennessee |

### University of Iowa—Dental Department.

The eighth annual commencement exercises of the Dental Department of the State University of Iowa took place at the Opera House, Iowa City, Iowa, on Monday evening, March 10th, 1890.

The annual address was delivered by Hon. Robert G. Cousins.

The number of matriculates for the session was one hundred and twenty.

The degree of D.D.S. was conferred on the following graduates by Charles A. Schaeffer, Ph.D , President of the University:

| NAME. | RESIDENCE. | NAME. | RESIDENCE. |
|---|---|---|---|
| T. G. Albin | Missouri | F. B. Kremer | Minnesota |
| J. V. Anderson | Pennsylvania | R. E. Lamoreux | Nebraska |
| F. J. Bethel | Colorado | F. H. Low | Iowa |
| A. D. Barker | Iowa | W. B. Mandeville | Minnesota |
| Benton Bement | New York | Edward Morton | Iowa |
| C. E. Booth | Wisconsin | W. F. McDonald | Iowa |
| C. M. Cobb | Iowa | Chas. R. McCandless | Iowa |
| C. E. Coleman | Iowa | W. E. Mabee | Iowa |
| G. W. Cook | Illinois | G. C. Marlow | Wisconsin |
| Charles Dorman | Iowa | E. H. Naumann | Iowa |
| Andrew Dingwell | Iowa | H. O. Rogers | Iowa |
| J. H. Dorival | Minnesota | G. W. Schwartz, M.D | Nebraska |
| F. E. Davoll | Dakota | S. L. Seeley | Iowa |
| J. W. Gluesing | Illinois | Richard Summa | Missouri |
| Nathaniel Glasgow | Iowa | W. H. Simpson | Iowa |
| C. H. Gibson | Minnesota | C. D. Tiffany | Iowa |
| R. H. Guy Huntley | Iowa | E. A. Taylor | Iowa |
| J. G. Hildebrand | Iowa | P. L. Van Winter | Washington |
| J. W. Hubbard | Iowa | H. Van Winter | Iowa |
| Harriet M. Jones | Iowa | T. B. Wallace | Iowa |
| W. H. Jallings | Minnesota | Hattie E. Wells | Iowa |
| Claude Kremer | Minnesota | | |

### Kansas City Dental College.

The eighth annual commencement exercises of the Kansas City Dental College were held at the Y. M. C. A. Auditorium, Kansas City, Mo., on Wednesday evening, March 12th, 1890.

The number of matriculates for the session was seventy-three.

The degree of D.D.S. was conferred on the following graduates by C. B. Hewitt, D.D.S., President of the Faculty:

| NAME. | RESIDENCE. | NAME. | RESIDENCE. |
|---|---|---|---|
| William S. Barker | Missouri | Clarence E. Johnson | Kansas |
| Alonzo M. Buckley | Arkansas | Shirley S. Millett | Missouri |
| James R. Campbell | Missouri | Wm. S. McDonald | Indiana |
| Frank L. Ewing | Kansas | Frank L. Overstreet | Kansas |
| Charles H. Gant | Nebraska | Henry J. Pfister | Kansas |
| Samuel M. Gant | Nebraska | Carl Ernest Sihler | Indiana |
| James E. Hawthorn | West Virginia | Charles R. Spruill | Illinois |
| Robert C. Hutcheson | Kansas | Wm. W. Williams | Missouri |

### Southern Medical College—Dental Department.

The third annual commencement exercises of the Dental Department of the Southern Medical College were held in connection with the medical department exercises at DeGive's Opera House, Atlanta, Georgia, on Wednesday evening, March 5th, 1890.

The number of matriculates for the session was sixty.

The degree of D.D.S. was conferred on the following graduates by Dr. Thomas S. Powell, President of the Board of Trustees:

| NAME. | RESIDENCE. |
|---|---|
| H. J. Arbey | Syria |
| T. F. Brannan | Georgia |
| W. F. Blassingame | Georgia |
| C. K. Chapman | Georgia |
| J. C. Cato | Georgia |
| J. R. Dedge | Georgia |
| J. T. Gordon | Georgia |
| A. L. Griffin | Georgia |
| C. W. Hendry | Georgia |
| J. H. Landry | Louisiana |
| E. J McIver | Georgia |
| C. H. Parrish | Georgia |
| D. Roberts | Georgia |
| W. S. Simmons | Georgia |
| E. G. Thomas | Georgia |

### Howard University—Dental Department.

The annual commencement exercises of the Dental Department of Howard University were held, in connection with those of the medical department, at the Congregational Church, Washington, D. C., on Friday evening, March 14th, 1890.

The address to the graduates was delivered by Prof. Charles B. Purvis, A.M., M.D., of the Faculty, and the annual address by J. E. Rankin, D.D., L.L.D., President of the University. The number of matriculates for the session was thirteen. The degree of D.D.S. was conferred on the following graduates of the dental class:

William M. Ash.

Andrew J. Brown.

Arthur T. Cooper.

Isaac C. Edington.

James H. Holsey.

Robert J. Macbeth.

Geo. A. Tompkins.

